# Natural Zeolite for The Purification of Saline Groundwater and Irrigation Potential Analysis

**DOI:** 10.3390/molecules27227729

**Published:** 2022-11-10

**Authors:** Timoth Mkilima, Davud Devrishov, Kydyrbekova Assel, Nurbala Ubaidulayeva, Almas Tleukulov, Alissa Khassenova, Nargiza Yussupova, Dinara Birimzhanova

**Affiliations:** 1 Department of Civil Engineering, L.N. Gumilyov Eurasian National University, Satpayev Street 2, Astana 010000, Kazakhstan; 2 Department of Immunology and Biotechnology, Moscow State Academy of Veterinary Medicine and Biotechnology, 23 Scryabin Str., 109472 Moscow, Russia; 3 Department of Management, L.N. Gumilyov Eurasian National University, Satpayev Street 2, Astana 010000, Kazakhstan; 4 Department of Chemistry and Chemical Technology, K. Zhubanov Aktobe Regional University, A. Moldagulova Ave. 34, Aktobe 030000, Kazakhstan; 5 Department of Water Resources and Reclamation, Kazakh National Agrarian Research University, Abaya Ave. 8, Almaty 050010, Kazakhstan; 6 Department of Geography and Environmental Sciences, Al–Farabi Kazakh National University, Al–Farabi 71, Almaty 050040, Kazakhstan; 7 Department of Chemistry, L.N. Gumilyov Eurasian National University, Satpayev Street 2, Astana 010000, Kazakhstan

**Keywords:** groundwater, salinity, sodium adsorption ratio, depth filter, natural zeolite

## Abstract

Groundwater is one of the main sources of water for irrigation used worldwide. However, the application of the resource is threatened by the possibility of high saline levels, especially in low-lying coastal regions. Furthermore, the lack of readily accessible materials for successful treatment procedures makes the purification of such water a constant challenge. Based on the fact that natural zeolite is one of the easily accessible and relatively cheap filter materials, this study examined the potential use of high-salinity groundwater filtered by natural zeolite for irrigation. Zeolite-filled filters at two different depths (0.5 m and 1 m) were studied. The samples were collected from the low-lying areas of Dar es Salaam City, Tanzania. The study observed that when the raw groundwater samples were exposed to the 0.5 m column depth, sodium (Na^+^) had the lowest removal efficiency at 40.2% and calcium (Ca^2+^) had the highest removal efficiency at 98.9%. On the other hand, magnesium (Mg^2+^) had the lowest removal efficiency, at about 61.2%, whereas potassium (K^+^) had up to about 99.7% removal efficiency from the 1 m column depth treatment system. Additionally, from the salinity hazard potential analysis, most of the samples fell within C4 (based on the electrical conductivity), which is a “very high salinity” class, and based on the quality it means the water cannot be directly applied for irrigation purposes. From the 0.5 m column depth, most of the samples fell within C3 (the “high salinity” class), and from the 1 m column depth most of the samples fell within C1 (“low salinity” class). The findings of this study offer some valuable insight into the prospective use of natural zeolite for the filtration of saline groundwater before its application for irrigation.

## 1. Introduction

Groundwater is a highly reliable source of water for many production activities in the world including irrigation. The phenomenon is also attributed to the fact that the global population is increasing, subjecting more pressure to the resource. That is to say that the global freshwater demand is also rising to meet rising global food consumption, which is expected to rapidly rise by 2050 to feed a projected global population [1]. As a result, one of the world’s most serious concerns is the state and management of freshwater resources [2]. Despite advances in water recycling technologies [3], groundwater will continue to provide the majority of global freshwater supplies in the coming decades. Among other production sectors, irrigation is categorized as the most important water use sector, accounting for roughly 70% of worldwide freshwater withdrawals and 90% of usage compared to other uses [4]. Globally, the saline intrusion has affected 1 billion people who rely on groundwater for drinking and irrigation. It is a symptom of a rising population, increasing groundwater use, rapid urban growth, and swelling demand for freshwater. Field studies are commonly used to determine the salinity of groundwater in aquifer systems [5].

It is also worth noting that irrigation is a key aspect of crop production in arid and semi-arid areas [6]. However, the irrigation water must not contain soluble salts in concentrations that are detrimental to plants or have a negative impact on the soil properties [7]. Unfortunately, most of the time, there is not enough water of this caliber to meet the needs of all the crops that are farmed [8]. Therefore, the purification of contaminated water for irrigation purposes becomes paramount to meet the water supply needs. However, finding relatively cheap and efficient treatment technology remains to be something of worldwide concern [9]. Therefore, it is highly significant to investigate and devise affordable and effective treatment technologies for achieving future sustainability. Mostly, farmers are forced to use irrigation water with high salt concentrations under these circumstances, which almost always results in lower crop yields [10]. Inappropriate use of this water can frequently result in crop failures and the development of saline or sodic soils, which then necessitate costly remediation to restore their productivity [11]. However, salt buildup starts to reduce crop yields long before any overt symptoms show.

Water enters plant roots by a process called osmosis, which is influenced by the amount of salt in the soil water and the water within the plant [12]. Water may flow from the plant roots back into the soil if the quantity of salts in the soil water is too high [13]. This causes the plant to become dehydrated, resulting in lower yields or possibly mortality [14]. Some ions (especially chloride) are harmful to plants, and when their concentration rises, the plant becomes poisoned and dies [15].

Moreover, as previously mentioned, food consumption has been sharply rising in the twenty-first century resulting from global population growth [16,17]; therefore, protecting farmland has become one of the most critical variables in food production [18]. It is estimated that close to 20% of the world’s farmland has salt-damaged soil with undesirable qualities for agriculture due to high-salinity water, and inexpensive desalination methods of high-salinity water are required to improve salt-damaged soil. One of the most popular techniques for treating salt water is reverse osmosis [19], which also has a somewhat high running cost. Other techniques include electrodialysis [20]. Reverse osmosis and electrodialysis both employ membranes. In the reverse osmosis and electrodialysis procedures, salt water travels through layers of membranes that, depending on the membrane charge, are selectively permeable to the salt water’s ions or impervious to them. An electric current provides the energy for the separation, which results in the production of brine in one section of the apparatus and fresh water in the other [21]. In such a context, devising efficient and cost-effective technologies to reduce salinity levels in groundwater before irrigation is of great importance. One of the potential approaches to achieve that is the application of natural zeolite as a depth filter. Natural zeolite is a cation exchanger that is relatively inexpensive and readily available [22,23,24]. Water, alkali, and alkaline earth-metal cations occupy pores in natural zeolites, which are hydrated aluminosilicate frameworks. These materials have outstanding adsorption properties due to their unique 3D porous structure. Because of its negatively charged surface, zeolite is a good cation exchanger in general [25].

In the literature, it is observed that natural zeolite materials have been applied in different other fields of water treatment including the removal of heavy metals from contaminated water [26,27]. Some other applications of natural zeolites recorded in the literature include municipal wastewater treatment [28]; textile wastewater [29,30]; as well as gray water treatment [31]. Moreover, it is very unfortunate that there is still no sufficient information attempting to highlight the potential of these adsorbent materials for the purification of saline groundwater from low-lying coastal areas to an irrigatable degree. It is also worth noting that the ability of natural zeolite to remove pollutants in any type of water can be highly limited to the characteristics of the water being treated [32], a phenomenon that has limited their application, especially in low-income communities.

This study investigated the potential applicability of high-salinity groundwater purified by natural zeolite based on the fact that it is among the readily available and relatively cheap filter materials. Two depth filters (0.5 m and 1 m) filled with zeolite were investigated. The samples were collected in different parts of Dar es Salaam. A number of analytical and statistical methods were used to investigate the problem.

## 2. Results and Discussion

### 2.1. Raw Water Characterization

Among the examined water quality parameters, sodium had a minimum concentration of 77 mg/L, a maximum concentration of 1616 mg/L, and an average concentration of 376 mg/L. In addition, it is important to note that sodium levels might affect irrigation water by possibly causing soil aggregates to scatter and crusts to form on the soil’s surface, which could prevent water from penetrating the soil [33]. The great solubility of sodium minerals accounts for sodium’s pervasiveness in the aquatic environment. The time of year, regional and local hydrological and geological factors, and salt consumption patterns all have a significant impact on sodium concentrations. Sodium concentrations in groundwater typically range from 6 to 130 mg/L [34].

Contrarily, calcium concentrations varied from 43 mg/L at the lowest end to 469 mg/L at the highest, with an average of 233 mg/L. In groundwater, calcium concentrations typically vary from 10 to 100 mg/L [35]. The principal sources of calcium are limestones and dolomites, which are carbonate rocks that have been dissolved by groundwater’s carbonic acid.

Magnesium concentrations were found to range from 21 mg/L at the lowest value to 243 mg/L at the highest, with 106 mg/L being the average. Magnesium concentrations in natural groundwater range from zero to around 50 mg/l, and rarely exceed 100 mg/L; therefore, calcium-based hardness typically predominates [36]. It is beneficial to have an appropriate concentration of both calcium and magnesium in the water because they are both essential plant nutrients in general [37]. Calcium and magnesium salts, however, may precipitate in the irrigation system if the water hardness is too high, damaging it or reducing its effectiveness [38].

Additionally, potassium concentrations were measured at a low of 8 mg/L, a maximum of 79 mg/L, and an average value of 42 mg/L. Total dissolved solids were measured with a minimum concentration of 5377 mg/L and a maximum concentration of 9845.5 mg/L, with an average value of 7680 mg/L. Electrical conductivity was also measured with a minimum concentration of 3114 μS/cm, a maximum concentration of 12,328 μS/cm, and an average concentration of 6286 μS/cm. It should be noted that measuring the electrical conductivity of irrigation water is a more convenient and indirect way to find out how much salt is present in it. The concentration of salt increases with conductivity [39]. As a result, a key measure for estimating the amount of dissolved salts in soil and water is electrical conductivity. Fresh groundwater typically has an electrical conductivity of less than 150 μS/cm. The average electrical conductivity measured in this study is 51.20 times higher than the average value for electrical conductivity in freshwater, or 5119.83% higher than the average typical value.

**Figure 1:**
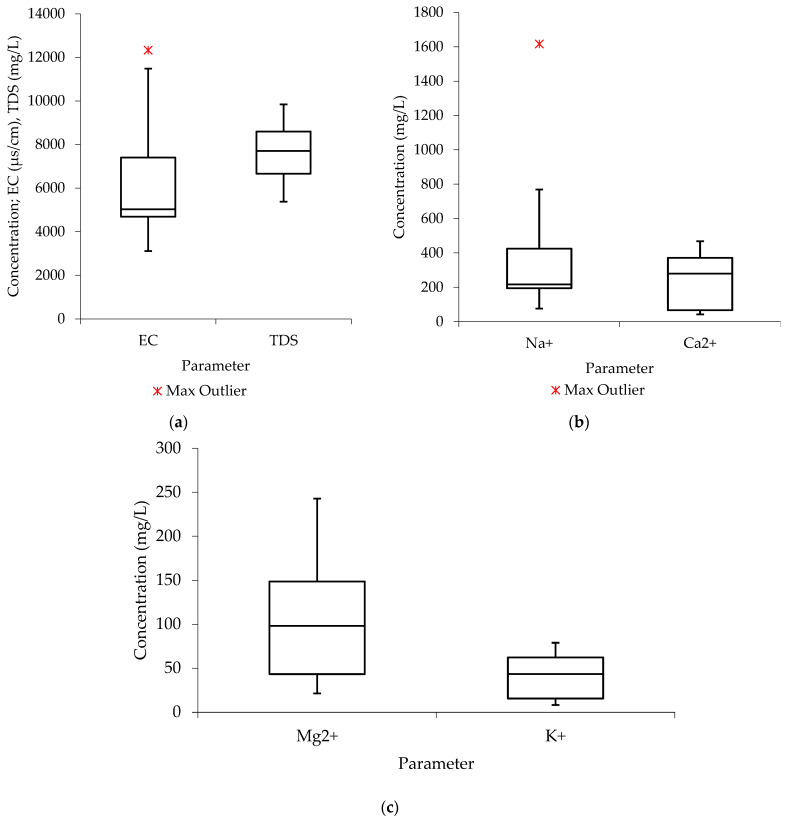
Data distribution before treatment (V_f_: 0.0035 L/s; pH: 6.4–12.5; T: 22.6–24.4 °C; *n* = 8) (**a**) electrical conductivity and total dissolved solids (**b**) sodium and calcium (**c**) magnesium and potassium.

**Figure 2:**
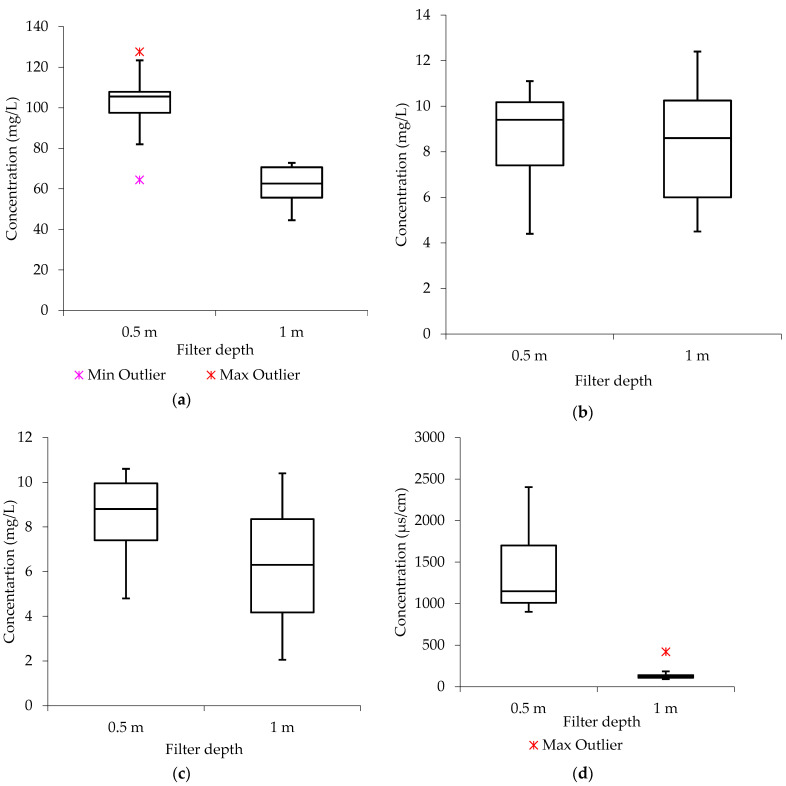

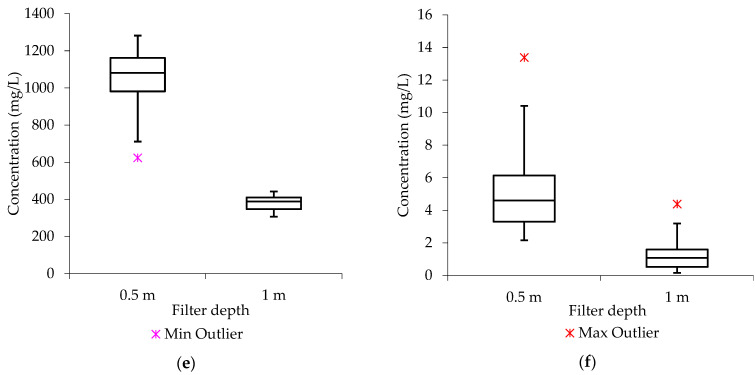
Data distribution after treatment (V_f_: 0.0035 L/s; pH: 6.4–12.5; T: 22.6–24.4 °C; *n* = 8) (**a**) sodium (Na^+^) (**b**) calcium (Ca^2+^) (**c**) magnesium (Mg^2+^) (**d**) electrical conductivity (EC) (**e**) total dissolved solids (TDS) (**f**) potassium (K^+^).

The findings of the data distribution analysis using the raw groundwater results are summarized in Figure 1. The distribution of the water quality data from the investigated parameters is regarded as being “negatively skewed” since the median in the boxplots from Figure 2a (TDS), Figure 2b (Ca^2+^), and Figure 2c (K^+^) is closer to the upper or top quartile. In the list of the analyzed samples, the data show a higher frequency of low-concentration values than high-concentration values. The median is seen to be more closely aligned with the lower quartile in Figure 2a (EC) and Figure 2b (Na^+^), respectively, indicating that the water quality data are positively skewed, with a larger frequency of high-concentration values than low-concentration values. Additionally, the median is seen to be nearer the center in the boxplots from Figure 1c (Mg^2+^), suggesting that the distribution of the water quality data is symmetric or normal.

### 2.2. Relationship between the Investigated Water Quality Parameters

In this part of the study, the correlation matrix was computed from sodium, calcium, magnesium, total dissolved solids, and pH. The selection of the investigated parameters is based on the fact that they have a relatively high potential of affecting irrigation systems. Major basic cations, also known as macronutrients, such as sodium, potassium, calcium, and magnesium, are geogenic solutes that primarily result from the weathering of rocks. Additionally, being close to the sea can raise the concentrations of these ions in the groundwater [40]. The correlation matrix aids in forecasting how the relationships between the variables will change over time. The correlation matrix gives a broad overview of the more or less significant relationship between various variables. It is an effective tool for compiling a sizable dataset and for locating and displaying data patterns. From Table 1, it can be seen that the highest correlation coefficient (0.966) was achieved from sodium and electrical conductivity. The phenomenon can be related to the fact that the conductivity rises with an increase in ion concentration because the ions in the solution carry the electrical current. As a result, conductivity rises when substances combine with water and split into ions. In that regard, more electrical conductivity is equivalent to more free ions [41,42]. To be more specific, due to their ability to move, ions in solutions can conduct electricity, which explains the strong correlation between sodium and electrical conductivity. When sodium (Na^+^) and chlorine (Cl^−^) combine to form sodium chloride in seawater, more electricity is conveyed, increasing the conductivity of the solution. In water, conductivity is caused by the transfer of electricity between ions. In plainer terms, conductivity rises in proportion to salinity [43]. In the study conducted by Yupeng et al. [44], on the total tissue sodium content at 3T/4T and quantitative conductivity mapping, a relatively high positive correlation was also observed between tissue conductivity and total sodium concentration with a *p*-value < 0.005.

TDS and sodium resulted in a correlation coefficient of 0.946, which also falls under the “very high” correlation group. Water’s total dissolved solids content is one of the main contributors to the particles and sediments that give it its color, flavor, and odor, as well as a general indicator of water quality. Therefore, the high correlation between total dissolved solids and sodium can be linked to the fact that total dissolved solids are the inorganic salts that are dissolved in water, mostly calcium, magnesium, potassium, sodium, bicarbonates, chlorides, and sulfates [45]. Therefore, an increase in sodium content in the water definitely increases the concentration of total dissolved solids.

Moreover, a high correlation coefficient (0.945) can also be observed between total dissolved solids and electrical conductivity. Conductivity, a metric of water’s capacity to carry an electric current, is directly related to the total amount of dissolved salts in the solution. This is due to the fact that as salts dissolve, positive and negative ions that can conduct an electrical current in proportion to their concentration. It should also be noted that water quality measures such as conductivity (EC) and total dissolved solids (TDS) are used to describe salinity levels. In the literature, the two parameters have been observed to be linearly correlating and can be expressed using Equation (1) [46].

(1) $$\mathrm{TDS}=k\times \mathrm{EC}\;\left(\mathrm{in}\;{25\;}^{{}^{\circ}}C\right)$$

where k is the proportionality constant.

More steps are involved in determining TDS from a water sample than in determining EC. TDS analysis is crucial since it can show groundwater quality, and because it allows us to understand the impact of seawater intrusion better than EC analysis. These factors make it interesting to conduct a study on the determination of TDS/EC ratios. From the EC result, the ratio value can be used to calculate the TDS concentration.

From Table 1, it can also be observed that pH correlated favorably with some of the parameters that were examined as determined by the correlation coefficients. In other words, the concentration of the parameters under investigation somewhat rose when pH was high.

On the other hand, most of the analyzed water quality parameters were found to be significantly correlated with one another, which is a phenomenon that can also be strongly related to their point of origin [47].

### 2.3. Purified Effluent Characterization

**Figure 3:**
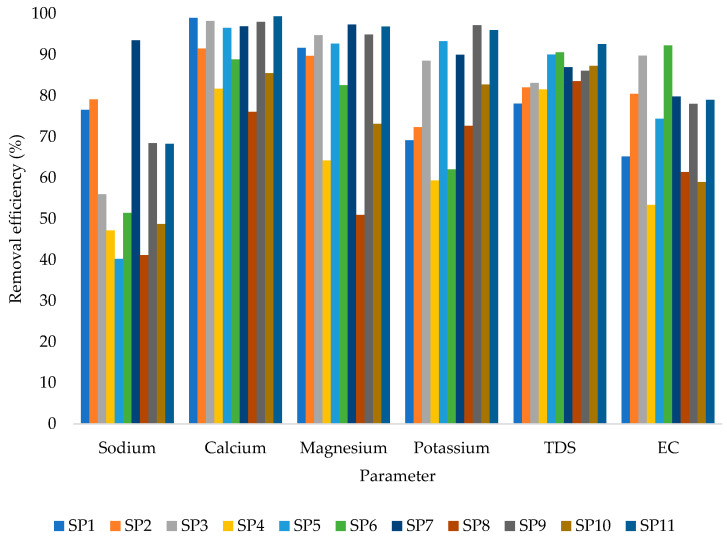
Removal efficiency from 0.5 m (V_f_: 0.0035 L/s; pH: 6.4–12.5; T: 22.6–24.4 °C; *n* = 8). SP1–SP11 are the sampling points (one to eleven).

Figure 2 presents the results of the data distribution analysis utilizing the processed groundwater results. Since the median in the boxplots from Figure 3 (Na^+^ (0.5 m), Ca^2+^ (0.5 m), Ca^2+^ (1 m), TDS (0.5 m), and TDS (1 m)) is closer to the upper or top quartile, the distribution of the water quality data from the analyzed parameters is characterized as being “negatively skewed.” The statistics demonstrate that there are more low-concentration values than high-concentration values in the list of evaluated samples. In Figure 3, for EC (0.5 m) and K^+^ (0.5 m), the median is observed to be more closely matched with the lower quartile, indicating that the water quality data are positively skewed, with a greater frequency of high-concentration values than low-concentration values. Additionally, the boxplots from Figure 3 for Na^+^ (1 m), Mg^2+^ (0.5 m), and Mg^2+^ (1 m) show that the median is closer to the center, indicating that the distribution of the water quality data is symmetric or normal.

### 2.4. Removal Efficiency

The results of the removal efficiency after the raw groundwater samples were subjected to the 0.5 m column depth are shown in Figure 3. Figure 3 shows that calcium had the highest recorded removal efficiency at 98.9% and sodium (Na^+^) had the lowest removal efficiency at 40.2%. On the other hand, the highest recorded Na^+^ removal efficiency from 0.5 m filter depth was 93.51%. It is important to note that ion exchange, adsorption, and salt storage are the major mechanisms controlling the process by which zeolites remove salt [48]. The ion exchange procedure is influenced by a number of variables, including the geochemical characteristics of zeolite, pH, co-existing anions, concentration, valency, surface charge, and experimental circumstances. The behavior of the Na^+^ ion adsorption isotherm on zeolites is composition-dependent and is generally claimed to follow either the Langmuir or Freundlich isotherm [49]. Most of the time, Na+ adsorption kinetics on zeolites are of an exothermic pseudo-second-order kind. To make the most of low-quality saline/sodic wastewater’s beneficial uses, sodium removal using zeolites seems to be an efficient water treatment technology.

A minimum removal efficiency from Ca^2+^ of 76.04% was observed, and a maximum removal efficiency of 99.38% was observed. It is crucial to emphasize that calcium is among the parameters leading to water hardness [50]. Calcium hardness is a measurement of the number of calcium ions in the water. Calcium and magnesium carbonates, bicarbonates, chlorides, and sulfates make up the majority of these minerals. In the study conducted by Hailu et al. [51], natural zeolite was used in the ion exchange process for groundwater to remove calcium, magnesium, and overall hardness; up to 80.2% removal efficiency was achieved for calcium.

From Mg^2+^, the minimum recorded removal efficiency was 50.97%, while the maximum removal efficiency was 97.37%. Natural zeolite has also been found to be quite effective at removing magnesium from water in the literature. For instance, a magnesium removal efficiency of up to 81.73% was attained in the work by Shahmirzadi et al. [52], who improved the removal and recovery of magnesium from aqueous solutions by employing modified zeolite.

Moreover, a minimum removal efficiency of 59.34% was recorded from K^+^, with 97.20% being the maximum recorded removal efficiency. Nevertheless, the removal efficiencies of TDS and EC ranged between 53.38% and 92.29%. Generally, the treatment system showed a relatively high removal performance for all the investigated parameters. In the literature, natural zeolite has also been observed to be highly effective in the removal of other contaminants apart from the ones investigated in this study. For instance, in the study conducted by Magalhães et al. [53], natural zeolite was reported to remove up to 96% of heavy metals and 90% of phosphoric compounds and was 96% effective for dyes, 80% effective for nitrogen compounds, and 89% effective for organics. It is also important to note that despite the fact that Cl^−^ removal was not a focus of the study, it remains one of the major challenges in treating water with natural zeolites. For example, in the study conducted by Takaaki Wajima et al. [54], it was reported that the natural zeolite performance for the removal of Cl^−^ was only 20% removal efficiency. High chloride levels in irrigation water or soil are hazardous to plants and may have an impact on how well they perform and how productive they are [55]. Therefore, it would be more appropriate to integrate the natural zeolite treatment system with other treatment approaches.

The more pores there are in the treatment media, the better the filtering effectiveness might be when it comes to filters. Because zeolite media have numerous pores, they not only catch particles between grains but also absorb them into their pores where they are then captured. The ability of the zeolite mineral to engage in cation exchange, whereby it absorbs positive ions from the water (such as dissolved metals, sodium, and ammonia) and exchanges them with other ions, contributes to this. Zeolite has a high pore density and a very effective surface area, allowing it to collect large quantities of pollutants without the need for backwashing. The adsorption process can be used by the media to catch and remove particles. Instead of passively becoming stuck between grains, particles cling to the surface of the media during this process, which is an active effect. According to conducted by Magalhães et al. [53], the authors reported that the removal efficiency of contaminants by zeolite varies depending on the substance to be removed and can reach up to 96% for the removal of contaminants in water.

The filter depth plays a significant role in the removal of physicochemical contaminants in water. Surface and depth filtration are the two methods used by mechanical filtering to remove particles. Surface filtration, a sieving technique that captures large particles on the top or leading surface of the filter, is used to eliminate many particles. Smaller particles are eliminated using depth filtering after passing through the surface layer. With depth filtration, smaller particles get caught as they pass through a filter’s progressively smaller pores. Finally, a process known as adsorption is used to remove the tiniest particles and dissolved molecules. During this process, particles are drawn to the surface of the filter medium and held there by weak electrical forces. For instance, in the study conducted by Yong et al. [56], that examined the performance of sand filtration system for polishing wastewater treatment with different sand bed depths (30 cm, 60 cm, and 90 cm), the highest total suspended solids and turbidity removal efficiencies of about 91.0% and 77.3%, respectively, were achieved from the sand depth of 90 cm.

**Figure 4:**
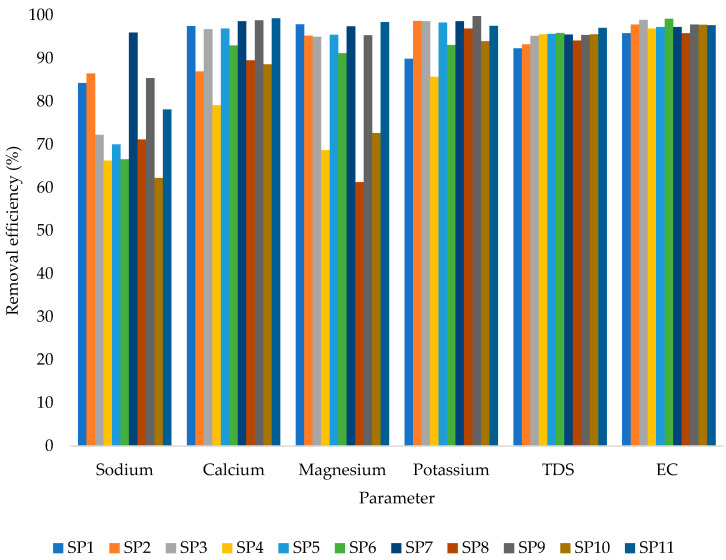
Removal efficiency from 1 m (V_f_: 0.0035 L/s; pH: 6.4–12.5; T: 22.6–24.4 °C; *n* = 8). SP1–SP11 are the sampling points (one to eleven).

Up to around 99.7% removal efficiency from the 1 m column depth treatment system was attained for potassium (K^+^), with magnesium (Mg^2+^) having the lowest removal efficiency of approximately 61.2%. Figure 4 reveals further that despite the relatively high removal efficiency of the treatment system, the removal of sodium was generally low compared to the other investigated water quality parameters. According to Karmen et al. [57], natural zeolite–clinoptilolite can achieve up to 100% removal efficiency for groundwater.

### 2.5. Analysis of Variance (ANOVA)

For a chosen set of water quality parameters (Table 2), a single-factor ANOVA was performed with an alpha value of 0.05 based on the characteristics of the raw wastewater and the treated effluent (0.5 m and 1 m column depths). In this part of the study, concentrations from each water quality parameter in the raw groundwater and purified effluent (0.5 m and 1 m) were subjected to the ANOVA. Then, the statuses of the generated *p*-values from the ANOVA were automatically computed using the built-in features of Microsoft Excel 2019. According to the findings of the analysis, there are statistically significant differences in terms of the concentrations in the examined water quality parameters between groups of data (raw wastewater, 0.5 m, and 1 m). Table 2 demonstrates that all the *p*-values were less than 0.05 (alpha value). The results of the analysis of variance also show that the applied treatment systems are effective because of the disparities between the concentration values of treated effluents and raw groundwater.

### 2.6. Tukey’s Honest Significance Test

When further examining the significance level of differences in terms of concentrations in the water samples using Tukey’s honest significance test, calcium and total dissolved solids were chosen as case studies. Table 3 shows that when the list of data from calcium in the effluent treated by the 0.5 m column depth was compared against the list of data from the raw groundwater, the difference produced a *p*-value of 0.001005, which is less than 0.01, making it statistically significant. Moreover, a *p*-value of less than 0.01 was retrieved when the list of data from raw groundwater was compared against the list of data from the treated effluent with 1 m column depth. However, when the list of data from 0.5 m column depth was compared against the list of data from 1 m column depth, a *p*-value of 0.899995 was retrieved, which is higher than 0.01, making the difference statistically insignificant. Similarly, the results of Tukey’s honest significance test continue to justify that the applied treatment systems are effective because of the disparities between the concentration values of treated effluents and raw groundwater; especially between the raw groundwater and the treated effluent using the 1 m column depth.

Similar to what was observed for calcium, Table 4 demonstrates that the difference between the data lists from total dissolved solids in the effluent treated by the 0.5 m column depth and the data lists from the raw groundwater yielded a *p*-value of 0.001005, which is less than 0.01, making it statistically significant. However, a *p*-value of 0.136821 was found when the list of data from the 0.5 m column depth was contrasted with the list of data from the 1 m column depth, making the difference statistically insignificant.

### 2.7. Salinity Hazard Potential Analysis

**Figure 5:**
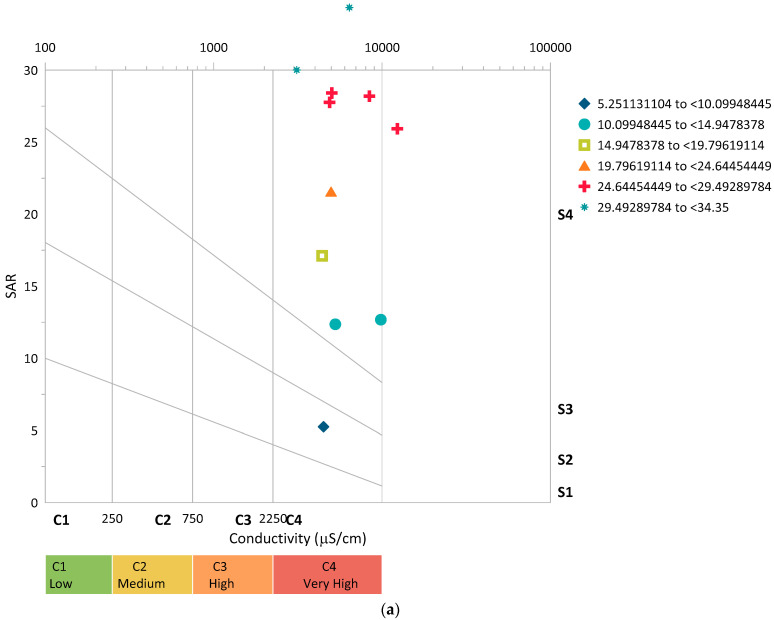

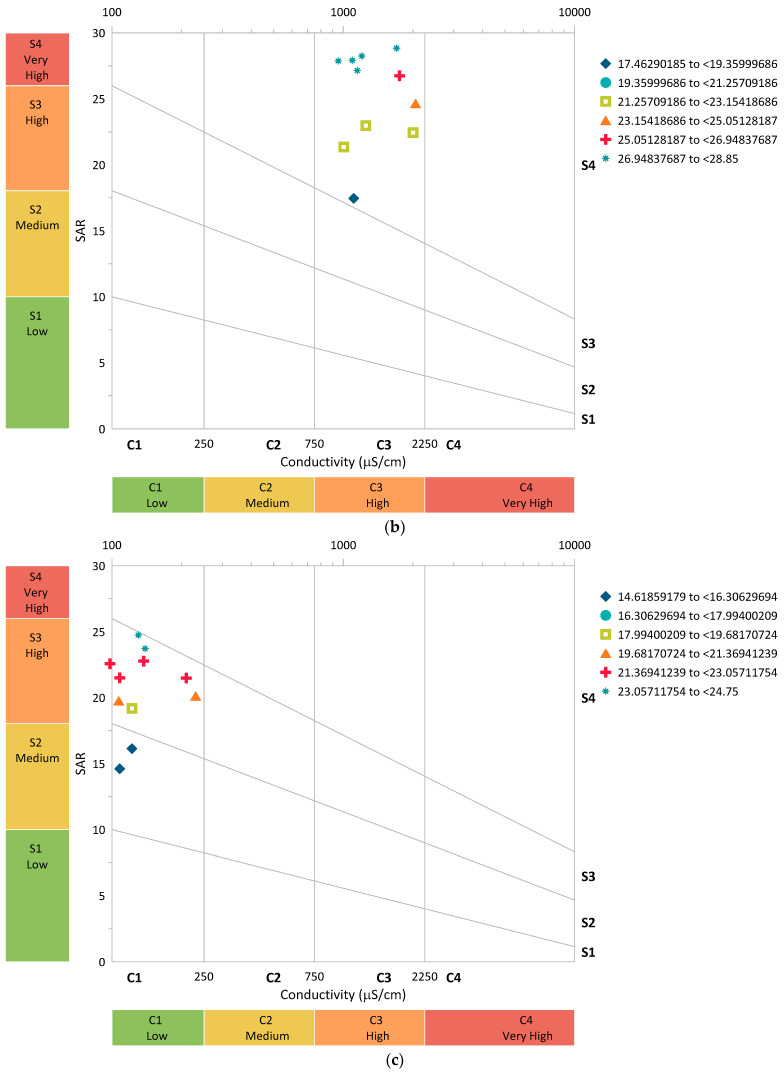
The salinity hazard potential from different water sources: (**a**) raw groundwater; (**b**) 0.5 m column depth; (**c**) 1 m column depth (V_f_: 0.0035 L/s; pH: 6.4–12.5; T: 22.6–24.4 °C; *n* = 8).

The investigation of the salinity hazard potential in the raw groundwater was one of the study’s most significant components. Salinity hazard potential analysis was then performed on the purified samples from the two treatment systems, as defined by the column depths, to assess whether they were suitable for irrigation. Wilcox diagrams were then used to condense the salinity hazard potential analysis data. The two most important variables used in the salinity hazard potential study were the sodium adsorption ratio and electrical conductivity. The salinity hazard potential analysis of the unprocessed groundwater is summarized in Figure 5a. Figure 5a shows that the majority of the samples from the untreated groundwater were within the C4S4 range. Based on the electrical conductivity, it means most of the samples fell within C4, which is a “very high salinity” class, and based on the quality it means the water cannot be directly applied for irrigation purposes. Such water has to be purified before being used for irrigation for most of the plant species in the world. Additionally, based on the SAR, most of the samples fell within S4, which is a “very high sodium hazard” class, and based on the quality it means the water is generally unsatisfactory for irrigation purposes. Normally, when the total amount of salts in the irrigation water is such that the salts build in the root zone to the point that crop yields are negatively affected, then there is a salinity problem related to water quality [58].

The salinity hazard potential analysis from the wastewater treated at the 0.5 m column depth is summarized in Figure 5b. Figure 5b shows that the majority of the samples from the raw groundwater fell within the C3S4 range. According to electrical conductivity, the majority of the samples fell into the “high salinity” C3 class, and according to water quality, the water can be used for irrigation with appropriate management techniques. Additionally, according to the SAR, the majority of the samples were classified as S4, or “extremely high sodium hazard,” which indicates that the water is typically unsuitable for irrigation. Still, such water has to be purified before being used for irrigation for most of the plant species in the world. The salinity hazard potential analysis from the wastewater treated at a 1 m column depth is summarized in Figure 5c. Figure 5c demonstrates that the majority of the samples from the untreated groundwater fell within the C1S3 range. Electrical conductivity indicates that the majority of the samples fell into the “low salinity” C1 classification, and water quality indicates that the water can be used right away for irrigation. SAR presents a small obstacle, with most samples falling in the S3 range and some in the S2 range. According to the classifications, the treated water can be utilized for irrigation in combination with some appropriate management measures.

## 3. Materials and Methods

### 3.1. Case Study Description

Dar es Salaam is the largest city in Tanzania located within −6.776012 latitude and 39.178326 longitude. The climatic conditions are defined mainly by dry and wet seasons; where June to October is regarded as the dry period of the year, with April and May being the wettest. In addition, the annual precipitation averages 1150 mm. In general, Dar es Salaam City is Tanzania’s largest urban area. In addition to being used as a primary source of water supply in the city, groundwater is also used to supplement surface water supplies. Dar es Salaam, however, is located in a coastal aquifer where salinity is one of many potential causes of contamination that might affect groundwater quality. There are currently limited groundwater quality data available to explain the level of pollution and impact of growing exploitation in the region. The case study was chosen because is one of the low-lying coastal areas with major problems from groundwater with excessive salinity. The utilization of groundwater as a source of water supply for Dar es Salaam City dates back to 1943. Though the majority of the city’s current boreholes were dug in 1997, during Tanzania’s catastrophic drought, groundwater use in Dar es Salaam has increased ever since. Now, more than 50% of the city’s people rely on groundwater [59]. For domestic, industrial, and water supply uses, there are currently more than ten thousand boreholes. The growing coastal population demands and reliance on groundwater need increased protection and management efforts for the resource.

### 3.2. Experimental Procedures, Raw Water Characteristics, and Filter Material Properties

Eleven boreholes were selected at random using purposeful sampling methodologies in order to evaluate the water quality for irrigation suitability in the research area, with a preference for those boreholes closest to the Indian Ocean coast. The boreholes were then assigned numbers between SP1 and SP11, and throughout the dry season, samples were obtained every week (once a week).

**Figure 6:**
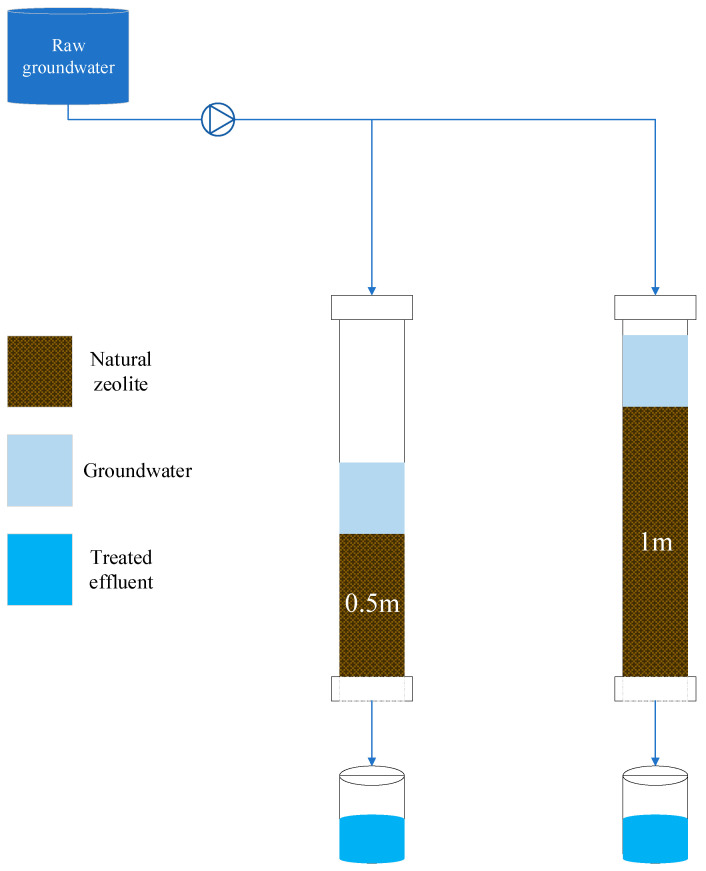
Experimental setup (V_f_: 0.0035 L/s; pH: 6.4–12.5; T: 22.6–24.4 °C; *n* = 8).

The obtained samples were then put through a purification process using natural zeolite, and two different fixed-bed columns with depths of 0.5 m and 1 m were used to study the impact of column depth on the treatment efficiency of the filter material (Figure 6). The polyvinyl chloride (PVC) column containers have a diameter of 5.08 cm. Both columns were filled with natural zeolite adsorbents (clinoptilolite), which have an average particle size of 1.5 mm and are composed of a microporous arrangement of silica and alumina tetrahedra (FM Stock and Supplies, Kenmare, Gauteng, South Africa).

To facilitate regular dispersion of flow within the columns, perforated plates with holes that were evenly spaced were placed over the top surfaces of the columns. A 100 L storage drum supplied the columns at a controlled rate of 0.0035 L/s. In actuality, the depth filter flow velocity was selected based on the depth filter’s average flow rates, working hours, and the incoming water’s available storage capacity. To keep all of the particles suspended, the wastewater was gently and constantly swirled. Wet packing was used on the porous substance to prevent stacking and air entrapment inside the file. The adsorbent bed was supported at the foot of each column by glass wool, and all three columns were positioned vertically. The packing of the column was followed by a period of running the column through with deionized water before the feed water was added. At predefined intervals, samples of the filtrate were collected. The experiments were all carried out at room temperature (20 to 25 °C).

Table 5 provides a summary of the physicochemical characteristics of the zeolite filter materials used in the study.

### 3.3. Analytical Procedures

In this study, six water quality parameters (EC, Na^+^, TDS, Mg^2+^, Ca^2+^, and K^+^) were studied. The selection of these factors (with the exception of potassium) is based on their importance in determining the suitability of groundwater for irrigation. The sodium-ion selective electrode method [60] was used to determine the levels of sodium in the water samples, while the ethylenediaminetetraacetic acid (EDTA) method [61], was used to determine both Mg^2+^ and Ca^2+^ concentrations in the groundwater samples, with Na2EDTA 0.05, Acetylacetone, and Tris(hydroxymethyl). The TDS Meter Digital Water Tester was also used to measure TDS and EC concentrations (Lxuemlu, Shenzhen, China). The potassium in the water samples was determined using the tetraphenylborate method. The lab pH meter (Frederick, MD, USA) was used for pH measurements. The water samples were analyzed in general according to the instructions in the American Public Health Association’s (APHA) standard methods for the examination of water and wastewater [62]. All of the samples were kept at 4 °C (for preservation) before being transported to the lab for examination and were analyzed the same day they were collected.

### 3.4. Statistical Methods

#### 3.4.1. Parameters’ Correlation

Correlation indices were computed for the analyzed parameters in this study. These indices were crucial in determining the strength of the association between the groundwater quality measures that were chosen. A high correlation, in general, showed that two or more variables had a strong association with each other according to the indices. A poor correlation indicated that the variables were not closely related. The classification of the correlation was as follows: 0–0.29 “weak”, 0.3–0.49 “moderate”, 0.5–0.69 “strong”, and 0.7–1 “very strong”.

#### 3.4.2. Data Distribution Analysis

The data distributions among the specified groundwater quality metrics were evaluated using box and whisker graphs. Data quartiles, also known as percentiles and averages, were used to evaluate data distribution on the basis of numerical data skewness.

#### 3.4.3. Analysis of Variance

Additionally, a single-factor analysis of variance (ANOVA) was employed in this investigation to determine whether or not the variations in the water quality data were statistically significant. The method evaluates the degree of variance within each group of water quality data using samples from each group. The difference between the *p*-values and alpha (0.05) values was used to compare the significance level. It is also important to note that the probability that you will reject the null hypothesis even if it is true is represented by the alpha value. The null hypothesis is accepted if the *p*-value is larger than the alpha value, whereas the *p*-value reflects the possibility of obtaining a result that is more extreme than the one you obtained from the experiment.

#### 3.4.4. Tukey’s Honest Significance Test

Tukey’s honest significance test, a single-step multiple comparison process and statistical test, was utilized in the investigation. It was used to determine whether there were any statistically significant differences between the means of the investigated parameters.

#### 3.4.5. Salinity Hazard Analysis

Both electrical conductivity and sodium adsorption ratios were judged based on distinct classes to investigate the salinity hazard potential. The salinity hazard zones were divided into four classes based on electrical conductivity: class one (C1), class two (C2), class three (C3), and class four (C4), from moderate salinity (C1) to very high salinity (C4). Table 6 summarizes the EC-based salinity hazard zones and their interpretations in terms of usability [63].

Table 7 summarizes the salinity hazard zones in terms of sodium adsorption ratio (SAR) and their usability implications. In the case of EC, the salinity hazard zones based on SAR were also divided into four classes and used in Wilcox diagrams: class one (S1), class two (S2), class three (S3), and class four (S4), with moderate sodium hazard (S1) to very high sodium hazard (S4) [64,65].

The 11 sampling sites were also used to create Wilcox diagrams. The “sodium hazard” (SAR) on the Y-axis was plotted against the “salinity hazard” (EC) on the X-axis to create the Wilcox diagram. It should be noted that the EC is by default shown in a log scale. Total salinity and sodium in relation to other ions are the main factors that determine whether water is suitable for irrigation. A Wilcox plot can be used to quickly establish whether water is viable or suitable for irrigation [66]. As a result, the diagrams were used to assess the hazard levels in the raw wastewater and the treated effluent.

## 4. Conclusions

The potential applicability of high-salinity groundwater purified by natural zeolite has been studied. It is important to note that zeolite is among the readily available and relatively cheap adsorbent materials. Two depth filters (0.5 m and 1 m) filled with zeolite were investigated. The samples came from Tanzania’s Dar es Salaam City’s low-lying neighborhoods. The study found that calcium (Ca^2+^) had the maximum removal efficiency at 98.9% and sodium (Na^+)^ had the lowest removal efficacy at 40.2% when the raw groundwater samples were subjected to the 0.5 m column depth. On the other hand, potassium (K^+^) had up to about 99.7% removal efficiency from the 1 m column depth treatment system, while magnesium (Mg^2+^) had the lowest removal efficiency, at about 61.2%. From correlation analysis, the highest correlation coefficient (0.966) was achieved for sodium and electrical conductivity. The phenomenon can be related to the fact that the conductivity rises with an increase in ion concentration because the ions in the solution carry the electrical current. Additionally, based on the findings from the ANOVA, it was observed that there are statistically significant differences in terms of the concentrations in the examined water quality parameters between groups of data (raw wastewater, 0.5 m, and 1 m) with *p*-values less than 0.01. The differences in concentration values between treated effluents and untreated groundwater demonstrate the effectiveness of the treatment systems used. Additionally, according to the salinity hazard potential study, the majority of the samples were classified as having a salinity level of C4 (extremely high salinity), which means they cannot be used for irrigation directly. Most of the samples fell into the C3 (“high salinity”) class from the 0.5 m column depth, and the C1 (“low salinity”) class from the 1 m column depth. We can infer from the study’s findings that direct groundwater use can have substantial detrimental effects on plant growth. However, relatively cheap materials such as natural zeolite can assist in purifying the raw groundwater to some degree, making it suitable for irrigation. Moreover, the removal of Cl- remains one of the main difficulties in using natural zeolite to clean water, despite the fact that it was not a focus of the study. Plants can be harmed by excessive chloride levels in irrigation water or soil, which may affect how effectively they function and how productively they grow. Therefore, combining natural zeolite treatment with additional therapeutic modalities would be more appropriate.

## Figures and Tables

**Table 1:** Correlation matrix from raw groundwater.

	EC	Na^+^	Mg^2+^	Ca^2+^	TDS	pH
EC	1					
Na^+^	0.966	1				
Mg^2+^	0.651	0.599	1			
Ca^2+^	0.837	0.766	0.726	1		
TDS	0.945	0.946	0.611	0.865	1	
pH	0.675	0.670	0.443	0.232	0.515	1

**Table 2:** Analysis of variance results.

Parameter	*p*-Value	Status (Is *p*-Value < 0.05?)	Definition
Calcium	4.16 × 10^−6^	TRUE	Statistically significant
Magnesium	1.75 × 10^−6^	TRUE	Statistically significant
Sodium	0.012003	TRUE	Statistically significant
Potassium	9.6 × 10^−7^	TRUE	Statistically significant
EC	1.07 × 10^−9^	TRUE	Statistically significant
TDS	8.27 × 10^−20^	TRUE	Statistically significant

**Table 3:** Tukey’s honest significance test results for calcium.

Treatments Pair	Tukey HSD Q Statistic	Tukey HSD *p*-Value	Tukey HSD Inference
Groundwater vs. 0.5 m	7.5942	0.001005	** *p* < 0.01
Groundwater vs. 1 m	7.61	0.001005	** *p* < 0.01
0.5 m vs. 1 m	0.0158	0.899995	insignificant

** statistically significant.

**Table 4:** Tukey’s honest significance test for total dissolved solids.

Treatments Pair	Tukey HSD Q Statistic	Tukey HSD *p*-Value	Tukey HSD Inference
Groundwater vs. 0.5 m	26.7366	0.001005	** *p* < 0.01
Groundwater vs. 1 m	29.5241	0.001005	** *p* < 0.01
0.5 m vs. 1 m	2.7876	0.136821	insignificant

** statistically significant.

**Table 5:** Physicochemical properties of the zeolite material used.

Parameter	Unit	Value
Particle size	mm	1.5
Bulk density	g/cm^3^	0.74
Particle density	g/cm^3^	1.4
Void ratio	%	48
Surface area	m^2^/g	42
Pore diameter	nm	0.7
Stability in terms of pH	-	neutral
Specific gravity	-	1.89

**Table 6:** Salinity hazard zones: based on EC.

EC Value (µS/cm)	Hazard Category	Interpretation
0–250 (C1)	Low salinity	Can be used safely
250–750 (C2)	Medium salinity	Can be used with moderate leaching
750–2250 (C3)	High salinity	Can be used for irrigation purposes with some management practices
2250–5000 (C4)	Very high salinity	Cannot be used for irrigation purposes

**Table 7:** Sodium hazard zones: based on SAR.

SAR Value	Hazard Category	Interpretation
0–10 (S1)	Low sodium hazard	Little or no hazard
10–18 (S2)	Medium sodium hazard	Appreciable hazard but can be used with appropriate management
18–26 (S3)	High sodium hazard	Unsatisfactory for most of the crops
>26 (S4)	Very high sodium hazard	Generally unsatisfactory for irrigation purposes

## Data Availability

The data that support the findings of this study are available from the corresponding author upon reasonable request.
